# The Elusive Nature of White Matter Damage in Anatomo-Clinical Correlations

**DOI:** 10.3389/fnhum.2012.00229

**Published:** 2012-08-03

**Authors:** Paolo Bartolomeo

**Affiliations:** ^1^INSERM, UMRS 975Paris, France; ^2^Department of Psychology, Catholic UniversityMilan, Italy

Molenberghs et al. (2012) contributed a clearly written meta-analysis on the debated issue of the anatomy of spatial neglect. They looked for a critical lesion site for neglect, and found several distinct regions whose damage has been associated with signs of neglect. Lesioned clusters were located in virtually the whole lateral surface of the right hemisphere (see their Figure 1), as well as in the white matter.

Molenberghs et al.’s study is timely and much needed after the recent publication of new evidence concerning this debate (see, e.g., Saj et al., 2012). Despite a fairly complete covering of the literature, however, a general methodological point prevents studies such as the present one from giving adequate weight to lesions to long-range white matter pathways.

As the authors acknowledge in the general discussion, while their method based on “the peak coordinates of the critical lesion site” is certainly appropriate for gray matter lesions, it seems problematic for long-range white matter bundles. At variance with gray matter lesions, where one can look for maximum overlap (Vallar and Perani, 1986) or analogous topological data (Bates et al., 2003), lesions in different sectors along a long-range white matter fascicle can produce similar effects by disconnecting the fascicle, independent of the precise location of the interruption (Catani and Mesulam, 2008). This is a general problem for studies looking for a “critical lesion site” in brain-damaged patients (Bartolomeo, 2011). Theoretically, similar behavioral deficits should be observed when a gray matter functional module is damaged as well as when white matter injury disconnects this module from the rest of the brain. Historically, neurologists have described neurological and neuropsychological deficits as disconnection syndromes (Geschwind, 1965; Catani and ffytche, 2005). However, the recent dominance of functional MRI (which identifies activation patterns in gray matter) and lesion symptom mapping based on gray matter injury (Bates et al., 2003) have led scholars to focus purely on the role of gray matter. Advances using techniques such as diffusion weighted imaging and the resulting tractography can help reveal the role of white matter (Catani, 2006). There is a clear need for methods that can integrate information from both gray and white matter injury, as these will likely provide better clinical significance and theoretical insight.

At present, the only way to explore the possibility that a deficit results from disconnection of a particular fascicle is to track the relevant fascicle, draw the lesions, and see whether or not they are located along the fascicle (see, e.g., Bourgeois et al., 2012). Methods based on this idea are being developed for group studies (Rudrauf et al., 2008). Concerning meta-analyses on neglect anatomy, Bartolomeo et al. (2007) mapped the hotspots defined by a small number of previous studies on fronto-parietal white matter, and found that the maximum lesion overlaps invariably occurred on or near the right superior longitudinal fasciculus (see also Doricchi et al., 2008; Thiebaut de Schotten et al., 2008). Such a procedure, however, would be much more cumbersome in larger meta-analyses such as the present one.

Despite these caveats, Molenberghs et al.’s results did reveal that “the largest region involved in the development of spatial neglect was a white matter lesion corresponding to the superior longitudinal fasciculus”, consistent with the meta-analysis of Bartolomeo et al. (2007). However, other white matter sites of damage might have been more difficult to pinpoint. For example, the possibility that a disconnection of the inferior fronto-occipital fasciculus (IFOF) may determine neglect in some patients (Urbanski et al., 2008) does not appear in the meta-analysis results. This might well depend on the relative rarity of such patients; however, it could also result from the fact that the IFOF is a particularly long white matter pathway; thus, IFOF-damaging lesions might be dispersed along its length and go undetected by methods based on lesion clustering.

To conclude, Molenberghs et al. (2012) made an important attempt to clarify a complex problem such as the anatomy of neglect. Their results, however, also highlight demanding methodological issues that need to be solved in future research.
